# From Acceptance to Dependence: Exploring Influences of Smart Healthcare on Continuous Use Intention of Mobile Health Services Among Older Adults with Chronic Illnesses in China

**DOI:** 10.3390/bs15010019

**Published:** 2024-12-28

**Authors:** Jiacheng Luo, Kewei Zhang, Qianghong Huang, Shan Jiang, Younghwan Pan

**Affiliations:** 1 Department of Smart Experience Design, Graduate School of Techno Design, Kookmin University, Seoul 02707, Republic of Korea; luojiacheng@kookmin.ac.kr (J.L.); zhangkewei@kookmin.ac.kr (K.Z.); huangqianghong@kookmin.ac.kr (Q.H.); jiangshan6633@kookmin.ac.kr (S.J.); 2 College of Literature and Arts Communication, Tongling University, Tongling 244061, China

**Keywords:** mobile health, older adults with chronic illness, continuous intention, TAM, VAM

## Abstract

With the acceleration of the aging process in China, chronic diseases have become one of the main health threats for older adults, creating significant pressure on society and the healthcare system. As information technology and artificial intelligence advance rapidly, smart health services have become readily accessible. However, utilization rates among the older adults, especially those with chronic illnesses, remain low, preventing them from fully benefiting from these advanced technologies. The value of mobile health (mHealth) services can only be realized through sustained use. Therefore, this study empirically investigates the continuous use intention of mHealth services from the perspective of older adults with chronic illnesses, integrating the Technology Acceptance Model (TAM) and Value-Based Adoption Model (VAM). A total of 372 questionnaires were collected from various cities in China, and data were analyzed using SPSS 24.0 and Partial Least Squares Structural Equation Modeling (PLS-SEM). Results indicate that perceived ease of use (*β* = 0.155, *p* = 0.004; *β* = 0.116, *p* = 0.027) and perceived usefulness (*β* = 0.175, *p* = 0.001; *β* = 0.151, *p* = 0.004) have a significant positive impact on attitude and perceived value. Perceived enjoyment significantly influences attitude (*β* = 0.147, *p* = 0.010), while perceived risk (*β* = −0.189, *p* < 0.001; *β* = −0.281, *p* < 0.001) and perceived cost (*β* = −0.155, *p* = 0.003; *β* = −0.130, *p* = 0.022) have a significant negative impact on attitude and perceived value. Both attitude (*β* = 0.357, *p* < 0.001) and perceived value (*β* = 0.314, *p* < 0.001) positively impact continuous intention. In total, only one of the twelve hypotheses was not supported. This study not only provides strong evidence for the effectiveness of the integrated TAM and VAM model in the mHealth field but also offers theoretical insights and practical recommendations for product optimization and promotion to mHealth service providers and designers.

## 1. Introduction

As healthcare technology advances, aging populations are rapidly increasing in developing countries. Due to the limited regenerative abilities of the older adults, they are more susceptible to chronic diseases. Statistics show that approximately 79.5% of older adults Chinese individuals aged 60 and above suffer from at least one chronic illness, nearly 50% have at least two, and over 25% live with three or more chronic conditions (Deng et al., 2014). This situation requires frequent hospital visits, which increases the burden on the health system (Hu & Wang, 2022). At the same time, there is an imbalance in the distribution of medical resources and an imbalance between supply and demand in health care, which has become a major obstacle to the development of health care in China (Ye et al., 2019).

Against this background, a new model of healthcare has emerged. Smart healthcare provides a new direction for medical practice by integrating technologies such as AI, IoT, and big data (Guan et al., 2023). Smart healthcare is defined as a technology-driven healthcare service approach that optimizes healthcare resource allocation, improves service efficiency, and reduces social healthcare costs (Jiang et al., 2017). Its features include digitization, personalization, and intelligence. As an integral part of smart healthcare, mHealth typically utilizes mobile devices such as smartphones, tablets, and patient monitors to deliver healthcare services. It is characterized by its broad coverage, high efficiency, and ease of use, making it a cost-effective and time-saving healthcare solution (Birkmeyer et al., 2021; Santos-Vijande et al., 2022). For instance, through mHealth services, users can easily book doctor appointments, receive test results, monitor health data in real time, and access personalized health management services. Consequently, the market size of mHealth has been rapidly expanding. Its importance has become increasingly prominent, particularly in the context of aging populations and the growing demand for chronic disease management.

Despite the many advantages of mHealth, continued use and adoption rates of mHealth services remain low among older adults with chronic illnesses. This trend hinders the development and adoption of mHealth (Meng et al., 2019). In China, even large mHealth companies with over 30 million registered users have only around 5% active users (Tian & Wu, 2022), with most older adults still relying on traditional healthcare services. Similarly, developing countries in a similar economic position to that of China are also facing this situation (Zhang & Zaman, 2020). Therefore, it is critical to understand the factors that influence the continued use of mHealth services by older adults with chronic conditions.

Through the literature, we find that most studies focus on explaining users’ technology adoption behavior. However, for older adults with chronic conditions, the value of mHealth services lies not only in helping them solve their health problems in the short term but also in providing ongoing support for chronic disease management, thereby improving their quality of life. Therefore, this study investigates mHealth continued adoption among older adults with chronic illnesses from both the perspectives of technology acceptance and value. The main aim is to explore how TAM factors (perceived usefulness, perceived ease of use, and usage attitude) and VAM factors (perceived cost, perceived risk, and perceived value) interact to influence older adults’ intentions to use mHealth services. (Back part shows all abbreviations). This research aims to fill a gap in the current literature by exploring factors influencing the ongoing use intentions of mHealth services among older adults managing chronic conditions. Additionally, the findings will provide valuable insights for mHealth platform developers, helping to enhance adoption rates among older adults with chronic illnesses and promoting the wider implementation of smart healthcare services.

The aim of this study is to understand the factors that influence users’ continued use of mHealth and the complex relationships between them and to provide theoretical and practical recommendations for the design and promotion of future mHealth services. The objectives of this study are as follows: (a) to develop and evaluate an integrated model combining TAM and VAM to understand factors influencing the continuous use of mHealth services among older adults with chronic illnesses; (b) to identify key factors affecting the use of mHealth services; and (c) to extract actionable insights that can guide the design and promotion of future mHealth services.

The structure of the paper is organized as follows: Section 2 reviews relevant theoretical frameworks and develops the study’s hypotheses. Section 3 details the research methodology and its main procedures. Section 4 provides an analysis of the data collected. Section 5 interprets the findings, while Section 6 addresses the study’s limitations and offers suggestions for future research directions. Finally, Section 7 summarizes the study.

## 2. Literature Review and Hypothesis Development

### 2.1. Mobile Health Services

In recent years, mobile health, as an emerging healthcare service model (Chomik & Piggott, 2015), has been rapidly transforming the traditional healthcare industry due to its convenience, ubiquity, and mobility (Rebolj & Menzel, 2004). mHealth services not only offer solutions to various health issues for remote areas, older adults, and individuals with limited mobility but also enable people to access medical services and health information conveniently and at a low cost (Bin Azhar & Dhillon, 2016; Wang et al., 2020).

With advancements in technology, m-health services have developed into a relatively mature system and play a crucial role in managing chronic diseases (Tian et al., 2019). Particularly among older adults, m-health can effectively manage diseases and improve their quality of life through services (Safdari et al., 2018) such as telemedicine, electronic health records (EHR), mobile health applications, disease detection, and prevention (Baudier et al., 2023; Kao & Liebovitz, 2017). In China, m-health services have rapidly progressed in areas such as telemedicine and online health management (Kao & Liebovitz, 2017). Many hospitals, in collaboration with technology companies, have developed applications that integrate health monitoring, diagnostics, and management. The convenience of mobile devices has enhanced the accessibility of healthcare services (Qudah & Luetsch, 2019; Vaitkienė et al., 2022), increased patient engagement (Singh et al., n.d.), and improved the efficiency of medical services (Eng & Lee, 2013; Karataş et al., 2022). Similarly, South Korea employs smart home healthcare services to monitor and manage personal health conditions (Kang et al., 2022), while Malaysia has numerous medical institutions utilizing electronic medical records to store and manage patient information (Enaizan et al., 2020). Consequently, m-health is regarded as an efficient healthcare solution (Gilbert et al., 2015).

However, Wong and Sa’aid Hazley (2021) highlighted that despite the numerous advantages of smart healthcare services, they still face significant challenges. Their successful implementation largely depends on the target population’s acceptance of the technology and service model (Liu & Tao, 2022). Previous studies have shown that smart healthcare services are not always embraced by users (Chen et al., 2022). This is particularly true for older adults with limited technological literacy, established living habits, and reduced cognitive abilities, who often encounter barriers when adopting and using m-health services. Such challenges hinder their ability to improve their health and limit their access to health technologies. Therefore, it is imperative to fully consider the needs and obstacles of older adults—such as technological usability; privacy protection; and user experience—and to delve into the key factors influencing the sustained usage intentions of older adults with chronic diseases. Addressing these factors can enhance the overall health and well-being of the older adults (Bin Azhar & Dhillon, 2016; Chen et al., 2022).

### 2.2. Older Adults’ Perceptions and Acceptance of Mobile Health Services

The older adults, as a key target group for chronic disease management, play a crucial role in the adoption and application of mobile healthcare technologies. Generally, the health conditions of older adults are often complex, with high prevalence rates of chronic diseases and an urgent need for health management. For instance, patients with chronic conditions such as diabetes, hypertension, and cardiovascular diseases require long-term health monitoring, personalized management plans, and emergency medical services (Li, 2013). The intelligent and real-time features of mobile healthcare technologies provide critical support for managing chronic diseases. For example, wearable health monitoring devices offer users real-time data, and smart applications deliver personalized management solutions tailored to individual needs.

Despite the technological advantages of mobile healthcare services, older adults still face numerous challenges in practical use (Jen & Hung, 2010; Meng et al., 2019), such as the complexity of technology operation, cost, and privacy concerns. Studies show that due to cognitive decline, the elderly often encounter difficulties in using mobile devices and adopting new technologies. Additionally, physical conditions such as weakened vision and hearing impairments make it inconvenient for them to use devices with small screens (Banskota et al., 2020; Wilson et al., 2021). Furthermore, economic cost is a significant factor affecting the older adults’ use of mobile healthcare services (Wilson et al., 2021). While many applications offer basic services for free, older adults may opt out due to additional service fees (Woldaregay et al., 2018). Moreover, data privacy and security issues remain major challenges for mobile healthcare applications, significantly impacting users’ trust (Baig et al., 2015; Newaz et al., 2021).

In recent years, scholars worldwide have extensively studied older adults’ acceptance of healthcare technologies and the factors influencing their continued use of mobile healthcare technologies (Davis, 1989). For example, surveys conducted on chronic disease patients in Iran revealed a positive attitude toward mobile healthcare technologies (Abbasi et al., 2020). Similarly, older adults in South Korea with multiple chronic conditions demonstrated a high willingness and acceptance of such technologies in healthcare (Ha & Park, 2020). Additionally, Hamine’s research highlighted the potential of mobile healthcare tools to better promote adherence to chronic disease management (Hamine et al., 2015). The above findings provide different explanatory perspectives on mHealth adoption behavior, but we found that existing studies focus more on technology acceptance, feasibility, etc. (Deng et al., 2014; Hoque & Sorwar, 2017) and less on continuance intention. However, the research suggests that continuance intention, as a more complex process, is more concerned with how users perceive the long-term value and reliability of the technology and persist in using the new technology after the initial adoption. It also determines the real impact and promotion value of an information system in older adults (Bhattacherjee, 2001). Therefore, this study will analyze the determinants affecting the intention to continue using mHealth services from the perspective of older adults with chronic diseases.

### 2.3. Technology Acceptance Model (TAM)

The TAM was introduced by Davis in 1989 (Davis, 1989; Davis et al., 1989), based on Fishbein and Ajzen’s (1977) Theory of Reasoned Action (TRA). TAM aims to explain users’ acceptance and intention to use new technologies or systems. It is one of the most frequently cited models for understanding individual behavior toward technology acceptance, extensively studied and validated (Chen et al., 2020; Shroff et al., 2011). The core constructs of TAM—Perceived Usefulness (PU) and Perceived Ease of Use (PEOU)—have repeatedly been shown as primary factors influencing technology acceptance. Consequently, TAM has been widely applied across various fields, including healthcare and assistive technologies, social networking, online shopping, the internet, computing, online public services, and entertainment. Table 1 summarizes prior empirical studies on TAM.

### 2.4. Value-Based Adoption Model (VAM)

The VAM was proposed by Kim et al. in 2007 (Han & Sa, 2022), building on Davis’s (Obaid, 2020) TAM and Zeithaml’s (Suhartanto et al., 2019) concept of perceived value. VAM is designed to understand the underlying motivations (both intrinsic and extrinsic) influencing users’ acceptance and intention to adopt specific technologies (Al-Rahmi et al., 2021). In VAM, Kim et al. represent perceived benefits through usefulness and enjoyment, while perceived sacrifice is represented by perceived costs and risks, establishing these as antecedents of perceived value, which is then analyzed to determine adoption intention (Navarro et al., 2021). Like TAM, VAM has also been applied across diverse fields, including e-commerce, mobile applications and digital services, healthcare technologies, social networks, online platforms, public services, and entertainment. Table 2 summarizes prior empirical studies on VAM.

### 2.5. Hypothesis Development

#### 2.5.1. Relationships Among Variables in TAM

In this study, perceived ease of use (PEOU) refers to the extent to which users perceive that mHealth is easy to operate and can be used quickly without much effort (Liu et al., 2005). Perceived usefulness (PU) refers to the extent to which users believe that mHealth can improve their quality of life, increase efficiency, and obtain more benefits (Al-Rahmi et al., 2019). Studies have shown that PEOU and PU directly influence users’ attitudes toward use, which in turn affects their behavioral intentions (Padilla-Meléndez et al., 2013). Hsiao and Tang (2015) confirmed that PEOU have a considerable positive impact on attitude (ATT) by exploring the acceptance of mHealth technology among older adults in Taiwan. Moores (2012) is in healthcare, validating the impact of PU on ATT. Based on this, the following hypotheses are proposed:

**H1:**  *PEOU of mHealth has a positive influence on ATT.*

**H2:**  *PU of mHealth has a positive influence on ATT.*

#### 2.5.2. Relationships Among Variables in VAM

Perceived Benefit (PB) refers to the positive value that users believe they gain from using mHealth technology or services, which can improve their quality of life, efficiency, or meet specific needs. It includes Perceived Usefulness (PU) and Perceived Enjoyment (PE). In VAM, PB is viewed as a key factor influencing adoption decisions. In this study, PE represents the degree of fun and pleasant experience users perceive while using mHealth; greater enjoyment motivates a stronger interaction (Chunxiang, 2014). Bian et al. (2023) confirmed that PU and PE have a significant positive effect on perceived value (PV) by examining the degree of adoption of Internet healthcare technology. Based on this, the following hypotheses are proposed:

**H3:**  *PU of mHealth has a significant positive impact on PV.*

**H4:**  *PE of mHealth positively influences PV.*

Perceived Sacrifice (PS) represents the various negative factors or costs users may face when using mHealth technology or services (Kim et al., 2017). It includes Perceived Cost (PC) and Perceived Risk (PR). In this study, PC refers to users’ perception of the sacrifices or losses associated with using the service (Zeithaml, 1988). This cost includes not only money but also time, energy, and other aspects (Chong et al., 2012). PR refers to the potential negative outcomes or losses users might face when using mHealth products or services and represents the users’ subjective assessment of potential negative impacts (Rapp et al., 2021). Studies by Bian et al. (2023) and Lin et al. (2020b) indicated that PC and PR had a significant negative effect on PV. Based on this, the following hypotheses are proposed:

**H5:**  *PC of mHealth has a significant negative correlation with PV.*

**H6:**  *PR of mHealth has a significant negative correlation with PV.*

#### 2.5.3. Integrated Relationships in TAM and VAM

According to Abdul-Halim’s research, PEOU has a significant impact on PU (Abdul-Halim et al., 2022). In VAM, perceived benefits encompass perceived usefulness. Sohn and Kwon’s research found a strong relationship between PEOU and perceived value (Sohn & Kwon, 2020). Based on this, the following hypothesis is proposed:

**H7:**  *PEOU of mHealth has a positive effect on PV.*

In the VAM, PE refers to the emotional pleasure users derive from using mHealth services. When users have an enjoyable interactive experience, it fosters positive feelings and enhances the appeal of the service (Marjerison et al., 2022). Research by Didyasarin et al. (2017) indicates that enjoyment significantly influences older adults’ attitudes toward mobile healthcare applications. Additionally, cost is an important factor affecting usage attitudes. High perceived costs often lead to resistance toward new technologies, resulting in more negative attitudes (Yang & Jolly, 2009). Furthermore, PR is considered a critical factor influencing users’ attitudes toward adopting and accepting new technologies (Wu et al., 2022). When users perceive a technology as high-risk, they are likely to form negative opinions about it (Mohd Rahim et al., 2022). Based on this, the following hypotheses are proposed:

**H8:**  *PE of mHealth has a positive effect on ATT.*

**H9:**  *PC of mHealth has a negative effect on ATT.*

**H10:**  *PR of mHealth has a negative effect on ATT.*

#### 2.5.4. Continuance Intention

In this study, continuance intention (CI) refers to the older adults’ willingness to continue using mHealth after initial adoption (Bhattacherjee, 2001). Hsieh et al.’s research indicates that usage attitude significantly influences CI (Hsieh et al., 2022). Users continue to use the mHealth service when they have a positive attitude towards it. Hsu et al. (2016) confirmed that ATT has a significant effect on CI by exploring the persistent intention to use telemedicine services by older adults. Based on this, the following hypothesis is proposed:

**H11:**  *ATT of mHealth has a positive effect on CI.*

PV refers to the fact that users arrive at an overall value judgment of mHealth services by comparing perceived benefits and sacrifices. Research has shown that perceived value has been consistently recognized as an important antecedent of adoption and continued use intentions (Li et al., 2018; Wang, 2008). When users perceive that mHealth services deliver health value, the willingness to continue using them is stronger (Boontarig et al., 2012). Wang and Cao (2023) confirmed that PV has a significant effect on CI by examining the willingness to continue using mHealth. Based on this, the following hypothesis is proposed:

**H12:**  *PV of mHealth has a positive effect on CI.*

**Figure 1:**
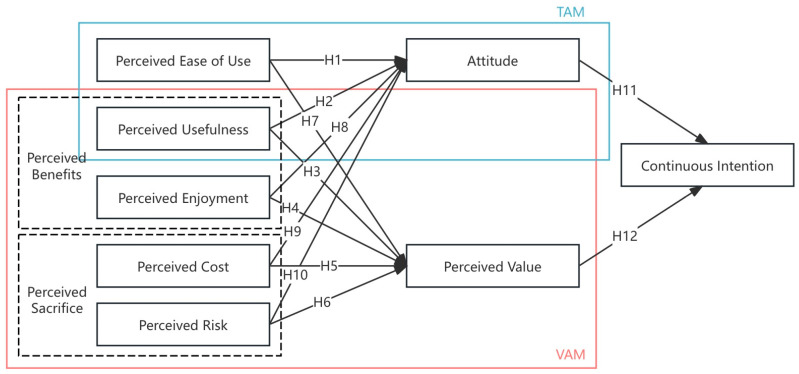
Theoretical Framework.

Therefore, this study constructs a research model based on TAM and VAM, where the independent variables include PEOU, PU, PE, PR, and PC, and they jointly influence the dependent variable CI through ATT and PV. The path hypotheses between the independent variables and the dependent variable are all included in the integrated theoretical model shown in Figure 1.

## 3. Methods

### 3.1. Questionnaire Design

The questionnaire for this study consists of three parts. The first part provides respondents with a brief overview of the study, emphasizing the voluntary nature of participation. All information will be recorded anonymously, and strict adherence to local data protection and storage policies will be maintained. The second part collects respondents’ basic demographic information, including gender, age, education level, living conditions, usage history, frequency of use, number of chronic conditions, and user experience. This section includes a screening question: “Have you used any mobile health-related services/applications”? If respondents answer “No”, the questionnaire terminates and is considered invalid. The third part aligns with the study’s model, with measurement items for each variable adapted from prior research to fit the context of mHealth. Specifically, the TAM constructs are adapted from Phang et al. and include PEOU (4 items), PU (4 items), and ATT (3 items). The VAM constructs are adapted from Kim et al. and include PE (4 items), PR (3 items), PC (3 items), and PV (4 items). CI (4 items) is adapted from Song et al.

To reduce cognitive load for older adults and prevent confusion or fatigue, all items are measured on a five-point Likert scale, ranging from “1 = Strongly disagree” to “5 = Strongly agree”. A preliminary test was conducted with 15 older adults over the age of 60 with chronic conditions (who were excluded from the main study) to evaluate the questionnaire’s validity and reliability. Minor adjustments were made based on their feedback. Details of the questionnaire items, definitions, and sources are provided in the accompanying Table 3.

Since all measurement items were initially written in English, translation professionals translated the questionnaire into the local language (Chinese) and performed back-translation to ensure the consistency of all items and expressions with the original meaning. From an academic ethics standpoint, all participants signed an informed consent form prior to completing the questionnaire, ensuring protection of their rights. They were clearly advised that the data collected would be solely used for academic research purposes, not for any commercial activities and that strict confidentiality would be maintained for all personal information.

### 3.2. Sampling and Data Collection

This study employed a quantitative research method to test the model, conducting a cross-sectional survey through a combination of online and offline channels between September and October 2024. The questionnaire data were collected from two main sources: first, 122 older adults aged 60–89 with chronic diseases were randomly invited to complete paper-based questionnaires at several hospitals in Hunan Province, China. Second, an online questionnaire was created through a professional survey platform in China, Questionnaire Star (https://www.wjx.cn/, accessed on 10 November 2024). A link and QR code were generated, and the survey was distributed to older adults aged 60 and above with chronic diseases via community staff using resident groups. Participation was voluntary, with no conflicts of interest involved throughout, and participants could withdraw at any time. This approach facilitated access to more older adults dispersed throughout the community. Snowball sampling can ensure the diversity and universality of questionnaire data (Naderifar et al., 2017). In addition, respondents can withdraw from the survey at any time while it is in progress.

A total of 415 questionnaires were distributed through both online and offline channels. After careful review by two researchers, 43 incomplete or unreliable responses were excluded, resulting in 372 valid responses. Hill stated that a sample size greater than ten times the number of measurement items for each construct is considered feasible in multivariate research, as suggested by Roscoe’s simple rule of thumb (Hill & Hamilton, 1998). Therefore, the sample size for this study meets the criteria for social science research, which is considered adequate sample size. As shown in Table 4.

### 3.3. Analysis Methods

Data analysis and model measurement for this study were conducted using SPSS 26.0 and Smart PLS 4.0. The PLS-SEM (Partial Least Squares Structural Equation Modeling) method was chosen for its advantages in handling multiple independent and dependent variables, addressing multicollinearity issues, and its flexibility regarding sample size, model identification, and data distribution assumptions (Hair et al., 2011). PLS-SEM is particularly suitable for predictive and exploratory research models. Hair et al. (2019) noted that PLS-SEM analysis involves several key stages: First, the fit of the structural model is assessed by standardized root mean square residuals (SRMR) and canonical fit index (NFI). Second, the fit of the structural model was measured by calculating metrics including factor loading, internal consistency reliability (e.g., Cronbach’s alpha and CR values), convergent validity (e.g., AVE), and discriminant validity (e.g., Fornell-Larcker criterion, HTMT, and cross-loadings) to measure the external model (i.e., the measurement model). Finally, the internal model (i.e., the structural model) was measured to validate the hypothesized relationships between model constructs. Consequently, this study uses the PLS-SEM model for data analysis, strictly following these guidelines and methodologies (Lin et al., 2020a).

## 4. Results

### 4.1. Model Fit

In structural modeling using PLS-SEM, two model fit indices were used to assess the model’s adequacy: the Standardized Root Mean Square Residual (SRMR) and the Normed Fit Index (NFI). SRMR is defined as the difference between observed correlations and the model-implied correlation matrix, with values below 0.08 indicating a good fit (Hair et al., 2019). NFI is an incremental fit measure, with values closer to 1 indicating a better model fit (Akram et al., 2022). In this study, the SRMR value was 0.034, and the NFI value was 0.893, suggesting that the model fit is relatively good, as shown in Table 5.

### 4.2. Common Method Bias

Common Method Bias (CMB) is described as systematic variance resulting from measurement methods and is a potential issue in all questionnaire items (Zhonglin, 2020). It is recommended to assess the Variance Inflation Factor (VIF) for all exogenous and endogenous constructs by using marker variables. According to Hair et al., when VIF ≤ 3.3, it indicates that the measurement model has no potential issues related to systematic variance (Leguina, 2015). In this study, all path VIF values ranged from 1.134 to 1.488, well below the threshold, suggesting that multicollinearity is not a concern in this research. As shown in Table 6.

### 4.3. Reliability and Validity Analysis

Reliability reflects the stability and consistency of the questionnaire outcomes, indicating the dependability of the measured data (Taherdoost, 2016). Validity, on the other hand, assesses the extent to which the measurement tools accurately reflect the true characteristics being measured, which is evaluated through factor analysis to verify the rationality of the research items (Bolarinwa, 2015). In this study, the reliability and validity of the questionnaire are evaluated to ensure the robustness of the data. We used PLS-SEM to conduct factor analysis, calculating Cronbach’s alpha, Composite Reliability (CR), and Average Variance Extracted (AVE). Generally, when Cronbach’s alpha and CR values reach 0.7, this indicates good internal consistency; values exceeding 0.8 signify high reliability (Fornell & Larcker, 1981). AVE, used to assess convergent validity, suggests strong validity when exceeding 0.5 (Bagozzi & Yi, 1988). Additionally, factor loadings show the correlation and consistency between observed variables and latent variables, with values above 0.7 indicating a strong association between them (Child, 2006).

In this study, Cronbach’s alpha and CR values (rho_a and rho_c) were all above 0.7, and AVE values exceeded 0.5, with factor loadings in each dimension also surpassing 0.7 (as shown in Table 7). The findings demonstrate that the measurement model possesses strong internal consistency, reliability, and convergent validity, thereby confirming the credibility of the study’s results.

To assess discriminant validity, which measures the independence among latent variables, we calculated the Fornell–Larcker criterion, HTMT, and cross loadings. As shown in Table 8, the square root of the AVE for each latent variable was greater than its correlations with other latent variables, indicating good discriminant validity (Fornell & Larcker, 1981). Additionally, as shown in Table 9, the HTMT values ranged from 0.317 to 0.548, well below the 0.85 threshold, further confirming strong discriminant validity (Hair et al., 2019). Furthermore, as shown in Table 10, each observed variable’s factor loading on its respective latent variable exceeded its loadings on other latent variables, also supporting discriminant validity.

In summary, these findings demonstrate that the model shows adequate fit, good reliability, and sufficient convergent and discriminant validity.

### 4.4. Hypothesis Testing

**Figure 2:**
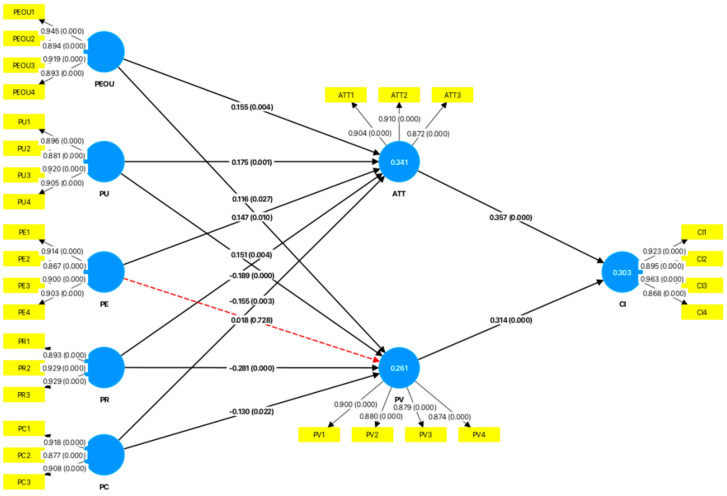
Results of the PLS structural model.

This study applied bootstrapping with repeated sampling, generating 5000 random samples to test path coefficients. As shown in Figure 2 and Table 11, PEOU positively affects ATT (*β* = 0.155, *p* = 0.004) and PV (*β* = 0.116, *p* = 0.027), supporting Hypotheses 1 and 7. PU has a significant positive impact on both ATT (*β* = 0.175, *p* = 0.001) and PV (*β* = 0.151, *p* = 0.004), validating Hypotheses 2 and 3. PE is significantly positively related to ATT (*β* = 0.155, *p* = 0.004), supporting Hypothesis 8. However, PE does not affect PV (*β* = 0.116, *p* = 0.027), so Hypothesis 4 is not supported. PR is significantly negatively related to both ATT (*β* = −0.189, *p* < 0.001) and PV (*β* = −0.281, *p* < 0.001), supporting Hypotheses 10 and 6. PC has a significant negative impact on both ATT (*β* = −0.155, *p* = 0.003) and PV (*β* = −0.130, *p* = 0.022), thus supporting Hypotheses 9 and 5. Additionally, both ATT (*β* = 0.357, *p* < 0.001) and PV (*β* = 0.314, *p* < 0.001) have a significant positive influence on CI, supporting Hypotheses 11 and 12.

### 4.5. Mediation Analysis

Previous literature suggests that using bootstrapping to compute the confidence intervals of indirect effects allows for determining significance, with indirect effects considered significant if the 95% confidence interval does not contain zero (Preacher & Hayes, 2008). As shown in Table 12, the confidence intervals for the relationships between PEOU, PU, PR, PC, and CI do not contain zero, indicating that ATT and PV have significant mediating effects. However, the PE confidence interval with PV includes zero, indicating no mediating effect.

To clarify and quantify the role of mediating variables between independent and dependent variables, we calculated the Variance Accounted For (VAF) value. Values between 20% and 80% suggest partial mediation, while values over 80% indicate full mediation. Results show that PEOU has a partial mediation effect on CI through ATT, explaining 59.78% and 39.13% of the variance. PU partially mediates the effect on CI through ATT, explaining 56.36% and 43.64% of the variance. PE fully mediates the effect through ATT, accounting for 89.66% of the variance. PR partially mediates the effect on CI through ATT, explaining 43.59% and 56.41% of the variance, and PC has a partial mediation effect, explaining 57.29% and 42.71% of the variance.

## 5. Discussion

This study constructs and validates an integrated model based on the TAM and VAM to explore key factors influencing the continued use of mHealth services among older adults in China with chronic illnesses. The empirical analysis reveals several key insights:

First, the results of this study indicate that PEOU, PU, PE, PR, and PC all have significant impacts on ATT. Among the positive influences, PU has the most significant effect, consistent with Moores’ findings (Moores, 2012). When users perceive that mHealth services can genuinely benefit them, their attitudes are significantly improved. Therefore, offering personalized chronic disease management features (e.g., real-time monitoring, health assessments) and integrating online and offline resources to enhance resource coherence can help users directly experience the benefits of the technology. Moreover, the influence of PEOU on user attitudes aligns with the findings of Hsiao and Tang (2015) Particularly when facing unfamiliar mHealth services, ease of use can alleviate users’ anxiety and foster positive attitudes. Simplifying design to reduce learning costs (e.g., large fonts, clear layouts), providing diverse options (e.g., language and gesture interaction), and ensuring operations align with users’ habits can enhance system friendliness. In contrast, the influence of PE on ATT is relatively smaller, consistent with the findings of Didyasarin et al. (2017) and Ho et al. (2019), but contrary to Alfany et al. (2019), who suggested that PE has no significant effect on ATT. This discrepancy may stem from differences in user group needs. Older adults with chronic diseases are more inclined to seek health management support through mHealth services. These services, by providing health data monitoring and online consultations, can offer a pleasant experience that significantly enhances their attitudes toward usage.

Among the negative influences, PR has the most substantial impact on ATT, as corroborated by prior studies (Mohd Rahim et al., 2022). High perceived risks weaken users’ attitudes toward usage due to concerns over issues such as data and privacy breaches or system failures. Platforms can address these concerns by strengthening management protocols (e.g., data encryption, two-factor authentication) and introducing government-certified evaluation mechanisms to ensure information security. Government agencies should also establish clear industry standards and privacy laws to strengthen regulation of mHealth services and protect user rights. Additionally, the negative impact of PC on ATT is relatively minor. This contradicts the findings of Zuelseptia et al. (2018), who reported that PC does not significantly affect ATT. This difference may be attributed to the nature of different user groups. Due to limitations in learning ability and physical condition, older adults are often more concerned about costs, as these not only include monetary expenses but also the additional time and effort required to learn and adapt to new technologies. This directly affects their usage attitudes. Platforms can optimize pricing structures and improve service efficiency to reduce the hidden costs of time and effort for users.

Secondly, the findings indicate that PEOU, PU, PR, and PC all have significant impacts on PV. Among the positive influences, PU has the most significant effect, supported by the studies of Bian et al. (2023) and Yu et al. (2017). For older adults, when mHealth services effectively meet their health management needs or provide useful health information, they tend to perceive the service as more valuable. Additionally, the impact of PEOU on PV aligns with the findings of Chairina (2021), as older adults are more likely to derive positive experiences from the ease of use, which translates into a high perception of service value. Providing clear and comprehensible services offers users instant assistance. Government agencies can promote community training programs to build trust in technology and boost confidence among older adults. However, this study found that PE does not have a significant impact on PV. This contrasts with studies that emphasize hedonic aspects as a source of value (Lee et al., 2013). The study suggests that mHealth services primarily focus on functionality and health support. For older adults with chronic illnesses, mHealth services are perceived more as practical tools rather than entertainment products; thus, the influence of PE on PV is less pronounced compared to entertainment applications. Based on this, future mHealth services could focus on optimizing user interfaces and functionality to reduce complexity and enhance ease of use. By offering rich and genuinely useful informational services, they can improve perceived usefulness. Additionally, incorporating elements of fun and interactivity can make the usage process more engaging, enabling users to experience the health benefits and convenience provided by these services. This approach can foster positive attitudes and perceived value, further driving the widespread and sustained adoption of mHealth services.

Among the negative influences, PR has the most significant impact on PV, consistent with the findings of Xie et al. (2021) and Bian et al. (2023). When mHealth services lack transparency in information security, accuracy, and reliability, users may worry about the misuse of personal health data, which significantly undermines their perception of service value, particularly among older adults. In contrast, the negative impact of PC on PV is relatively minor, aligning with the findings of Jen and Hu (2003). Older adults are particularly sensitive to costs, including money, time, and effort. In high-cost scenarios, their perceived value can decrease significantly. Therefore, future mHealth services should prioritize privacy protection and data security. Additionally, by simplifying operational processes and reducing usage costs (e.g., lowering fees and optimizing time efficiency), these services can minimize users’ psychological and practical costs, thereby enhancing their confidence and satisfaction in using the services.

Finally, the results indicate a strong positive correlation between ATT, PV, and CI among older adults with chronic conditions. This suggests that older adults’ intention to continue using mHealth services is significantly strengthened by their positive attitudes and perceived high value of the services. Among these factors, ATT has the greatest influence on CI, consistent with the findings of Hsu et al. (2016). Attitude plays a critical role in shaping users’ confidence and dependence on services, particularly in the healthcare domain. When older adults hold a positive attitude toward mHealth services, their emotional and cognitive evaluations significantly influence their behavioral decisions, thereby increasing the likelihood of continued use. Additionally, the significant positive impact of PV on CI aligns with the findings of Wang and Cao (2023). The more value older adults perceive in mHealth services, the more likely they are to use them long-term. Therefore, future mHealth service designs should focus on enhancing user experience and increasing perceived value. This can be achieved by offering personalized health services, improving service reliability, and enhancing convenience, thereby fostering positive user attitudes and dependence on the services. Ultimately, these efforts can effectively promote the continued intention of older adults.

## 6. Implications and Limitations

### 6.1. Implications

The value of mHealth services for older adults with chronic conditions lies in their long-term use. While prior studies have focused primarily on the functional design and advantages of mHealth, this study examines the key factors influencing continued mHealth service use from the perspective of older Chinese users with chronic conditions. This research validates the efficacy of an integrated model based on TAM and VAM in this field, offering theoretical support for future studies. The findings provide developers with practical recommendations for enhancing older adults’ continued usage intentions, thus meeting their health management needs.

On a theoretical level, TAM and VAM are widely recognized theories, but each has limitations. By integrating TAM and VAM, this study provides a multidimensional perspective and expands the model’s applicability. It also highlights the bridging role of ATT and PV in continued usage intention among older adults. By exploring the interrelations between these variables, this study offers theoretical support for health technology acceptance among older adults.

On a practical level, this study offers concrete guidance for designing and promoting mHealth services. First, enhancing the actual utility and ease of use of mHealth services can effectively boost users’ trust and reliance. Additionally, addressing perceived risk and cost is crucial, as these factors significantly impact attitudes and perceptions. mHealth service providers should offer a secure platform to protect users’ private information.

This study also offers theoretical and practical guidance to promote mHealth services among older patients with chronic conditions, improve user engagement, optimize service design, alleviate pressure on medical resources, and enhance long-term health management and support for chronic disease patients.

### 6.2. Limitations and Future Research

This study has certain limitations. First, the sample was limited to older chronic disease patients in China, so the findings may not be generalizable to other age groups or cultural backgrounds. Expanding the geographic scope of the sample in future studies could enhance generalizability. Additionally, since most data were collected online, there may be some interpretation biases among respondents. Future studies should increase the offline sample size to improve data reliability. Finally, this study lacks a long-term dynamic observation and cannot examine behavioral changes over time. Future research should consider longitudinal study designs to explore trends and drivers of user behavior and intention.

## 7. Conclusions

This study developed and evaluated an integrated model based on TAM and VAM to investigate key factors influencing continued mHealth usage among older patients with chronic conditions in China. A total of 372 responses were collected both online and offline for data analysis, covering eight factors and twelve hypotheses, of which eleven were supported, confirming the integrated relationships within the theoretical model. Notably, PR has the largest negative impact on both ATT and PV, reflecting older adults’ concerns over privacy, security, and reliability. Many older adults worry about privacy breaches and information security issues, which can lead to distrust or even aversion to mHealth services. Reducing perceived risks is thus a key focus for future improvement. Finally, these findings provide valuable insights for mHealth service development: emphasizing user experience and functionality, strengthening privacy and data security, lowering risk, increasing trust, enhancing long-term usage intentions, and promoting the widespread application of health management services.

## Figures and Tables

**Table 1:** Previous empirical research on TAM models.

Model Theory	Population/Size	Objective	Results	Reference
TAM	Older adults (220)	IoT-based healthcare services	Usefulness significantly influences Korean older adults ‘intention to use IoT-based healthcare services. Professional support and personalization have no significant impact on usefulness, whereas interaction and convenience significantly impact usefulness.	(Hong et al., 2020)
TAM	Older adults (204)	Mobile healthcare services	Perceived ease of use has a positive impact on perceived usefulness and adoption intention. Technology anxiety and dispositional resistance to change influence both perceived ease of use and resistance to change. Resistance to change does not influence perceived ease of use or adoption intention.	(Guo et al., 2013)
TAM + Four Contextual Constructs	General users (279)	Mobile chronic disease management system (MCDMS)	Perceived ease of use and perceived usefulness positively influence users’ adoption intentions. for MCDMS. Perceived disease threat and initial trust positively impact adoption intention. Perceived risk and technology anxiety negatively impact perceived ease of use.	(Zhu et al., 2018)
TAM + Perceived Irreplaceability/Perceived Credibility/Compatibility/Social Influence	Older adults with chronic diseases (223)	Digital health wearable devices	Perceived ease of use and perceived usefulness positively influence continued use of digital health wearable devices. Perceived irreplaceability, perceived credibility, compatibility, and social influence all positively affect continuance intention.	(Ahmad et al., 2020)
TAM + TAM2	General users (101)	Telemedicine	Perceived ease of use and perceived usefulness positively impact intention to use telemedicine. Job relevance and result demonstrability influence both perceived ease of use and perceived usefulness. Experience does not significantly impact perceived usefulness or intention to use.	(Su et al., 2020)

**Table 2:** Previous empirical research on VAM models.

Model Theory	Population/Size	Objective	Results	Reference
VAM	healthcare professionals (268)	Mobile platform of medical and senior care (MPMSC)	Perceived value and legal concerns predict healthcare professionals’ intention to adopt MPMSC. Outcome expectations, perceived mobility, perceived effort, and privacy concerns predict perceived value.	(Xiong & Zuo, 2023)
VAM + Employee Burnout	Older adults (12031)	Mobile healthcare services	Perceived usefulness, perceived enjoyment, and perceived complexity are positively correlated with perceived value. Perceived value directly and positively impacts adoption intention and negatively impacts employee burnout. Employee burnout is negatively correlated with adoption intention.	(Bian et al., 2023)
VAM + UTAUT2	General users (100)	Smart health wearable devices	Perceived privacy and perceived fee have a significant negative impact on perceived value. Perceived enjoyment, perceived expectancy, and effort expectancy have a significant positive impact on perceived value. Perceived value positively influences intention to use. Perceived trust negatively impacts intention to use.	(Niknejad et al., 2020)
VAM + UTAUT2	General users (287)	Smart health wearable devices	Perceived fee, perceived enjoyment, perceived expectancy, effort expectancy, social influence, facilitating conditions, perceived trust, and perceived health increase are key factors for using SWWs. Facilitating conditions do not significantly influence intention to use. Perceived privacy also has no effect on perceived value.	(Niknejad et al., 2022)
VAM + Usage Experience	General users (518)	Smart product services	Usefulness, flexibility, fee, and technicality as dimensions of perceived value determine adoption intention, which further influences actual use.	(Yu & Sung, 2023)

**Table 3:** Questionnaire scales and references.

Variable	Definition	Items	Questions	Reference
Perceived Ease of Use (PEOU)	The degree to which users find mHealth services easy to understand and use.	4	PEOU1. Learning to use mHealth services is easy for me. PEOU2: I can quickly become proficient in using mHealth services. PEOU3: My interaction with mHealth services is clear and easy to understand. PEOU4: Overall, mHealth services are easy to use.	(Davis, 1989; Deng, 2013; Phang et al., 2006)
Perceived Usefulness (PU)	Users’ expectations regarding the utility of mHealth services in helping manage their health.	4	PU1: I believe mHealth services can improve my healthcare efficiency. PU2: I think mHealth services are beneficial to my health. PU3: I believe mHealth services make my life more convenient and efficient. PU4: I think mHealth services will help me manage my health more effectively.	(Deng, 2013; Dhagarra et al., 2020; Phang et al., 2006)
Perceived Enjoyment (PE)	The sense of enjoyment and satisfaction users feel when using mHealth services.	4	PE1: I enjoy interacting with mHealth services. PE2: I feel happy using mHealth services. PE3: I find it interesting to obtain health information through mHealth. PE4: I enjoy using mHealth to manage my health.	(Agarwal & Karahanna, 2000; Hasan et al., 2020; Kim et al., 2007)
Perceived Risk (PR)	Users’ concerns about potential privacy breaches or technical issues when using mHealth services.	3	PR1: I feel safe when using mHealth services. PR2: I am not concerned that mHealth will disclose my health data. PR3: I am not worried about unauthorized use of my personal information when using mHealth.	(Kim & Kim, 2004; Liao et al., 2022; Wu & Wang, 2005)
Perceived Cost (PC)	The costs users may incur when using mHealth services.	3	PC1: I do not need to spend additional financial resources (e.g., mobile network fees and equipment) to use mHealth services. PC2: I feel that using mHealth does not require a significant amount of my time. PC3: I feel that using mHealth does not demand much effort from me.	(Davis, 1989; Luarn & Lin, 2005; Wang & Wang, 2010)
Attitude (ATT)	Users’ overall evaluation of mHealth services.	3	ATT1: I have a positive attitude toward using mHealth services. ATT2: Using mHealth services encourages me to manage my health more actively. ATT3: I believe that using mHealth services is a good choice.	(Deng et al., 2014; Venkatesh et al., 2003)
Perceived Value (PV)	Users’ perception of the overall utility and value provided by mHealth services.	4	PV1: Compared to the effort I need to invest, using mHealth services is beneficial to me. PV2: Compared to the time I need to spend, using mHealth services is worthwhile. PV3: Compared to the sacrifices I need to make, using mHealth services is valuable. PV4: Overall, using mHealth services has brought me good value.	(Kim et al., 2007; Reiter et al., 2017; Sirdeshmukh et al., 2002)
Continuance Intention (CI)	Users’ willingness to continue using mHealth services.	4	CI1: I intend to continue using mHealth services in the future. CI2: I plan to use mHealth services to manage my health in the future. CI3: I expect to increase my usage of mHealth services in the future. CI4: I would recommend mHealth services to others.	(Ashfaq et al., 2020; Bhattacherjee, 2001; Song & Jo, 2023)

**Table 4:** Participant demographic information (n = 372).

Category	Item	Frequency	Percent
Gender	Male	203	54.57%
	Female	169	45.43%
Age	60–64	77	20.70%
	65–79	142	38.17%
	70–74	87	23.39%
	75–79	45	12.10%
	80+	21	5.65%
Education level	Middle school or below	69	18.55%
	High school	141	37.90%
	Vocational college	117	31.45%
	Bachelor’s degree	35	9.41%
	Graduate degree or above	10	2.69%
Number of people living together with	1	183	49.19%
	2	121	32.53%
	3+	68	18.28%
Usage history	<1 month	22	5.91%
	1–6 months	40	10.75%
	6–12 months	70	18.82%
	1–3 years	126	33.87%
	>3 years	114	30.65%
Usage frequency	At least once week	130	34.95%
	At least once month	70	18.82%
	At least once every three months	64	17.20%
	At least once every six months	68	18.28%
	Other	40	10.75%
Suffers from several chronic diseases	3 or less	214	57.53%
	4 or more	158	42.47%
Usage experience	Yes	372	100%

**Table 5:** The results of the construct assessment.

Fit Index	Computed Value	Reference
SRMR	0.034	(Hair et al., 2019)
NFI	0.893	(Akram et al., 2022)

**Table 6:** The results of the construct assessment.

Path	VIF
PEOU → ATT	1.377
PEOU → PU	1.377
PU → ATT	1.458
PU → PV	1.458
PE → ATT	1.488
PE → PV	1.488
PR → ATT	1.322
PR → PV	1.322
PC → ATT	1.433
PC → PV	1.433
PV → CI	1.134
ATT → CI	1.134

**Table 7:** Reliability and validity analysis.

	Variable	Factor Loading	AVE	CR (rho_a)	CR (rho_c)	α
PEOU	PEOU1	0.945	0.833	0.934	0.952	0.933
	PEOU2	0.894				
	PEOU3	0.919				
	PEOU4	0.893				
PU	PU1	0.896	0.811	0.923	0.945	0.922
	PU2	0.881				
	PU3	0.920				
	PU4	0.905				
PE	PE1	0.914	0.803	0.926	0.942	0.919
	PE2	0.867				
	PE3	0.900				
	PE4	0.903				
PR	PR1	0.893	0.841	0.920	0.941	0.906
	PR2	0.929				
	PR3	0.929				
PC	PC1	0.918	0.812	0.891	0.928	0.884
	PC2	0.877				
	PC3	0.908				
ATT	ATT1	0.904	0.802	0.877	0.924	0.876
	ATT2	0.910				
	ATT3	0.872				
PV	PV1	0.900	0.780	0.909	0.934	0.906
	PV2	0.880				
	PV3	0.879				
	PV4	0.874				
CI	CI1	0.923	0.834	0.934	0.952	0.933
	CI2	0.895				
	CI3	0.963				
	CI4	0.868				

**Table 8:** Discriminant validity (Fornell–Larcker criterion).

	ATT	CI	PC	PE	PEOU	PR	PU	PV
ATT	0.895							
CI	0.465	0.913						
PC	−0.411	−0.499	0.901					
PE	0.417	0.480	−0.478	0.896				
PEOU	0.402	0.452	−0.341	0.329	0.913			
PR	−0.416	−0.465	0.336	−0.417	−0.337	0.917		
PU	0.430	0.477	−0.396	0.379	0.466	−0.347	0.901	
PV	0.344	0.437	−0.332	0.293	0.331	−0.424	0.361	0.883

**Table 9:** Discriminant validity (HTMT values).

	ATT	CI	PC	PE	PEOU	PR	PU	PV
ATT								
CI	0.513							
PC	0.467	0.548						
PE	0.462	0.517	0.529					
PEOU	0.445	0.485	0.376	0.356				
PR	0.461	0.504	0.373	0.455	0.367			
PU	0.479	0.515	0.437	0.412	0.502	0.378		
PV	0.385	0.473	0.366	0.317	0.358	0.463	0.394	

**Table 10:** Discriminant validity (cross loadings).

	ATT	CI	PC	PE	PEOU	PR	PU	PV
ATT1	0.904	0.430	−0.351	0.388	0.332	−0.426	0.383	0.303
ATT2	0.910	0.417	−0.377	0.368	0.381	−0.382	0.367	0.287
ATT3	0.872	0.402	−0.376	0.364	0.367	−0.305	0.406	0.335
CI1	0.412	0.923	−0.468	0.482	0.431	−0.447	0.434	0.401
CI2	0.458	0.895	−0.472	0.433	0.403	−0.417	0.413	0.403
CI3	0.419	0.963	−0.451	0.457	0.423	−0.434	0.431	0.401
CI4	0.405	0.868	−0.427	0.378	0.394	−0.399	0.463	0.389
PC1	−0.384	−0.464	0.918	−0.438	−0.306	0.314	−0.371	−0.340
PC2	−0.357	−0.425	0.877	−0.386	−0.300	0.265	−0.312	−0.244
PC3	−0.368	−0.457	0.908	−0.465	−0.316	0.326	−0.384	−0.307
PE1	0.387	0.419	−0.446	0.914	0.283	−0.375	0.330	0.277
PE2	0.323	0.403	−0.411	0.867	0.297	−0.326	0.323	0.202
PE3	0.398	0.413	−0.411	0.900	0.273	−0.397	0.335	0.267
PE4	0.381	0.482	−0.446	0.903	0.329	−0.391	0.370	0.295
PEOU1	0.372	0.425	−0.299	0.315	0.945	−0.298	0.444	0.314
PEOU2	0.365	0.408	−0.319	0.267	0.894	−0.308	0.448	0.296
PEOU3	0.369	0.420	−0.302	0.317	0.919	−0.327	0.390	0.285
PEOU4	0.361	0.397	−0.326	0.303	0.893	−0.298	0.419	0.314
PR1	−0.324	−0.399	0.297	−0.375	−0.312	0.893	−0.289	−0.326
PR2	−0.378	−0.447	0.317	−0.374	−0.318	0.929	−0.329	−0.435
PR3	−0.431	−0.430	0.309	−0.399	−0.299	0.929	−0.332	−0.395
PU1	0.381	0.420	−0.365	0.327	0.406	−0.294	0.896	0.340
PU2	0.389	0.445	−0.346	0.368	0.446	−0.306	0.881	0.302
PU3	0.398	0.425	−0.358	0.357	0.433	−0.326	0.920	0.336
PU4	0.380	0.427	−0.357	0.315	0.395	−0.324	0.905	0.322
PV1	0.340	0.418	−0.339	0.264	0.323	−0.383	0.338	0.900
PV2	0.320	0.402	−0.296	0.253	0.314	−0.376	0.315	0.880
PV3	0.290	0.340	−0.251	0.235	0.225	−0.365	0.314	0.879
PV4	0.261	0.377	−0.281	0.282	0.300	−0.373	0.309	0.874

**Table 11:** Analysis of pathway relationships.

Hypothesis	*β*	SD	*t*-Value	*p*	CIs (2.5–97.5%)	Result
PEOU → ATT	0.155	0.054	2.860	0.004	(0.048; 0.265)	Supported
PEOU → PV	0.116	0.052	2.216	0.027	(0.013; 0.219)	Supported
PU → ATT	0.175	0.052	3.390	0.001	(0.078; 0.280)	Supported
PU → PV	0.151	0.052	2.892	0.004	(0.048; 0.252)	Supported
PE → ATT	0.147	0.057	2.583	0.010	(0.038; 0.260)	Supported
PE → PV	0.018	0.053	0.348	0.728	(−0.086; 0.121)	Unsupported
PR → ATT	−0.189	0.052	3.671	0.000	(−0.292; −0.089)	Supported
PR → PV	−0.281	0.050	5.604	0.000	(−0.379; −0.182)	Supported
PC → ATT	−0.155	0.052	2.950	0.003	(−0.257; −0.052)	Supported
PC → PV	−0.130	0.057	2.286	0.022	(−0.243; −0.021)	Supported
ATT → CI	0.357	0.051	7.046	0.000	(0.253; 0.453)	Supported
PV → CI	0.314	0.051	6.206	0.000	(0.215; 0.415)	Supported

**Table 12:** Analysis of pathway relationships.

Hypothesis	*β*	SD	*t*-Value	*p*	CIs (2.5–97.5%)	Result	VAF
Direct effect							
PEOU → CI	0.092	0.027	3.345	0.001	(0.039; 0.146)	Supported	-
PU → CI	0.110	0.028	3.883	0.000	(0.058; 0.168)	Supported	-
PE → CI	0.058	0.029	2.002	0.045	(0.003; 0.118)	Supported	-
PR → CI	−0.156	0.027	5.702	0.000	(−0.212; −0.103)	Supported	-
PC → CI	−0.096	0.029	3.269	0.001	(−0.156; −0.042)	Supported	-
Indirect effect							
PEOU → ATT → CI	0.055	0.021	2.608	0.009	(0.017; 0.098)	Supported	59.78%
PEOU → PV → CI	0.036	0.018	1.978	0.048	(0.004; 0.077)	Supported	39.13%
PU → ATT → CI	0.062	0.021	3.030	0.002	(0.025; 0.106)	Supported	56.36%
PU → PV → CI	0.048	0.019	2.524	0.012	(0.014; 0.088)	Supported	43.64%
PE → ATT → CI	0.052	0.022	2.378	0.017	(0.013; 0.098)	Supported	89.66%
PE → PV → CI	0.006	0.017	0.339	0.735	(−0.025; 0.043)	Unsupported	10.34%
PR → ATT → CI	−0.068	0.022	3.117	0.002	(−0.114; −0.029)	Supported	43.59%
PR → PV → CI	−0.088	0.021	4.172	0.000	(−0.135; −0.051)	Supported	56.41%
PC → ATT → CI	−0.055	0.022	2.531	0.011	(−0.101; −0.017)	Supported	57.29%
PC → PV → CI	−0.041	0.020	2.045	0.041	(−0.083; −0.006)	Supported	42.71%

## Data Availability

The data presented in this study are available on request from the corresponding author.

## Abbreviations

The following abbreviations are used in this manuscript:

WHO	World Health Organization
mHealth	Mobile health
TAM	Technology acceptance model
VAM	Value-based adoption model
TRA	Theory of Reasoned Action
PLS-SEM	Partial least squares structural equation modeling
PEOU	Perceived ease of use
PU	Perceived usefulness
PE	Perceived enjoyment
PB	Perceived benefit
PS	Perceived sacrifice
PR	Perceived risk
PC	Perceived cost
ATT	Attitude
PV	Perceived value
CI	Continuance intention
SRMR	Standardized Root Mean Square Residual
NFI	Normed Fit Index
CMB	Common Method Bias
VIF	Variance Inflation Factor
CR	Composite Reliability
AVE	Average Variance Extracted
HTMT	Heterotrait-Monotrait ratio of correlations
